# Supplementary material for: To Be, or Not to Be: That Is the Hamletic Question of Cryptic Evolution in the Eastern Atlantic and Mediterranean Raja miraletus Species Complex
Source: Animals (Basel). 2023 Jun 28;13(13):2139. doi: 10.3390/ani13132139 (PMC10339953; doi:10.3390/ani13132139)
Supplement: Supplementary file 1 [file animals-13-02139-s001.zip › Text S1.pdf]

animals

MDPI

To be, or not to be: that is the Hamletic question of cryptic evolution in the Eastern Atlantic and Mediterranean *Raja miraletus* species complex

Alice Ferrari [1], Valentina Crobe [1], Rita Cannas [2], Rob W. Leslie [3], Fabrizio Serena [4], Marco Stagioni [5], Filipe O. Costa [6], Daniel Golani [7], Farid Hemida [8], Diana Zaera-Perez [9], Letizia Sion [10], Pierluigi Carbonara [11], Fabio Fiorentino [4,12], Fausto Tinti [1,*] and Alessia Cariani [1]

[1] Department of Biological, Geological and Environmental Sciences, University of Bologna, 40126 Bologna, Italy; alice.ferrari6@unibo.it (A.F.); valentina.crobe2@unibo.it (V.C.); alessia.cariani@unibo.it (A.C.)
[2] Department of Life and Environmental Sciences, University of Cagliari, 09126 Cagliari, Italy; rcannas@unica.it
[3] Branch Fisheries Management, Department Agriculture, Forestry and Fisheries, Cape Town 8018, South Africa; roblesliesa@hotmail.com
[4] Institute for Biological Resources and Marine Biotechnology, National Research Council, 91026 Trapani, Italy; fabrizio.serena@irbim.cnr.it (F.S.); fabio.fiorentino@irbim.cnr.it (F.F.)
[5] Laboratory of Marine Biology and Fisheries, Department Biological, Geological and Environmental Sciences, University of Bologna, 61032 Fano, Italy; marco.stagioni3@unibo.it
[6] Centre of Molecular and Environmental Biology (CBMA) and ARNET-Aquatic Research Network, Department of Biology, University of Minho, Campus de Gualtar, 4710-057 Braga, Portugal; fcosta@bio.uminho.pt
[7] Department of Evolution, Systematics and Ecology, The Hebrew University of Jerusalem, Jerusalem 9190401, Israel; dani.golani@mail.huji.ac.il
[8] Ecole Nationale Supérieure des Sciences de la Mer et de l'Aménagement du Littoral, Campus Universitaire de Dely Ibrahim, Algiers 16320, Algeria; hemidafarid@yahoo.fr
[9] Institute of Marine Research, 5817 Bergen, Norway; diana.zaera-perez@hi.no
[10] Department of Biosciences, Biotechnologies and Environment, University of Bari Aldo Moro, 70125 Bari, Italy; letizia.sion@uniba.it
[11] COISPA Technology and Research, 70126 Bari, Italy; carbonara@coispa.it
[12] Stazione Zoologica Anton Dohrn, 90149 Palermo, Italy
* Correspondence: fausto.tinti@unibo.it
† These authors contributed equally to this work.

Supplementary Material

Text S1

Molecular methods

DNA isolation

Total genomic DNA (gDNA) was extracted from about 20 mg of tissue using the Invisorb® Spin Tissue Mini Kit (Stratec®molecular) following manufacturer's protocol (http://www.stratec.com/en/molecular/Products_Molecular/Genomic_DNA/Invisorb_Spin_Tissue_Mini_Kit/Invisorb_Tissue_Mini.php).

PCR amplification

mtDNA *COI* sequence

From the extracted gDNA, a fragment of the mitochondrial Cytochrome Oxidase subunit I (*COI*) gene of about 650 bp was amplified using the *COI*-3 primer cocktail described by Ivanova et al. [61]. The PCR reactions were performed in 50 μL total volume

containing 4 μL of pure gDNA corresponding to ~25 ng, 10 μL of buffer (1X), 5 μL of MgCl2 (2.5 mM), 0.5 μL of dNTP mix (0.1 mM each), 1 μL of each primer (0.2 mM) and 0.25 U of Taq DNA Polymerase. All PCR reagents were supplied by Promega®, except for primers, supplied by LifeTechnologies®. The cycle was planned with the following thermal profile: 94°C for 2 mins, 35 cycles of 94°C for 30 secs, 52°C for 40 secs, 72°C for 1 min and a final extension step at 72°C for 10 mins and it was performed on a Biometra T-Gradient Thermocycler. The PCR products were electrophoresed on agarose gel at 2 % and amplicons were stored at -20ºC until shipping to Macrogen Europe b.v. (Amsterdam, the Netherlands). Individual Sanger sequencing was carried out on Applied Biosystems 3730xl DNA Analyser.

Expressed Sequence Tag-linked microsatellite loci

Eight *EST-SSRs* developed from the little skate *Leucoraja erinacea* [58] and cross-amplifying in several skate species [56,62] were optimised for cross-amplification in the *R.* [*miraletus*] species complex. The *EST-SSR* PCR reactions were performed in a 10 μL total volume containing 3 μL of gDNA corresponding to ~20 ng, 2 μL of PCR Buffer (1X), 0.5 μL of $MgCl_2$ (1.25 mM), 0.8 μL of dNTP mix (0.05 mM each), 0.5 μL of each primer (0.5 μM; 0.25 μM for LERI 26) and 1 U of Taq DNA Polymerase. All PCR reagents were supplied by Promega®, except the primers which were from LifeTechnologies®. DNA amplifications were run on a Biometra T-Gradient Thermocycler as follows: after an initial denaturation at 94°C for 3 mins, amplification was performed with 30 cycles consisting of denaturation at 94°C for 30 secs, annealing at 53°C for 30 secs, extension at 72°C for 30 secs, followed by a final extension at 72°C for 10 mins. Amplicons were electrophoresed on 2.5% agarose gel and stored at -20ºC until shipping to Macrogen (South Korea). Individual genotyping was performed on ABI3100 Genetic Analyser (Applied Biosystems), using labelled forward primers and LIZ HD500 (Applied Biosystems) as internal size standard.

References

56. Frodella, N.; Cannas, R.; Velonà, A.; Carbonara, P.; Farrell, E.; Fiorentino, F.; Follesa, M.; Garofalo, G.; Hemida, F.; Mancusi, C.; et al. Population connectivity and phylogeography of the mediterranean endemic skate *Raja polystigma* and evidence of its hybridization with the parapatric sibling *R. montagui*. *Mar. Ecol. Prog. Ser.* **2016**, *554*, 99–113, doi:10.3354/meps11799.

58. El Nagar, A.; McHugh, M.; Rapp, T.; Sims, D.W.; Genner, M.J. Characterisation of polymorphic microsatellite markers for skates (Elasmobranchii: Rajidae) from expressed sequence tags. *Conserv. Genet.* **2010**, *11*, 1203–1206, doi:10.1007/s10592-009-9919-8.

61. Ivanova, N.V.; Zemlak, T.S.; Hanner, R.H.; Hebert, P.D.N. Universal primer cocktails for fish DNA barcoding. *Mol. Ecol. Notes* **2007**, *7*, 544–548, doi:10.1111/j.1471-8286.2007.01748.x.

62. Catalano, G.; Crobe, V.; Ferrari, A.; Baino, R.; Massi, D.; Titone, A.; Mancusi, C.; Serena, F.; Cannas, R.; Carugati, L.; et al. Strongly structured populations and reproductive habitat fragmentation increase the vulnerability of the Mediterranean starry ray *Raja asterias* (Elasmobranchii, Rajidae). *Aquat. Conserv. Mar. Freshw. Ecosyst.* **2022**, *32*, 66–84, doi:10.1002/aqc.3739.
