# Supplementary figures and images for: To Be, or Not to Be: That Is the Hamletic Question of Cryptic Evolution in the Eastern Atlantic and Mediterranean Raja miraletus Species Complex
Source: Animals (Basel). 2023 Jun 28;13(13):2139. doi: 10.3390/ani13132139 (PMC10339953; doi:10.3390/ani13132139)

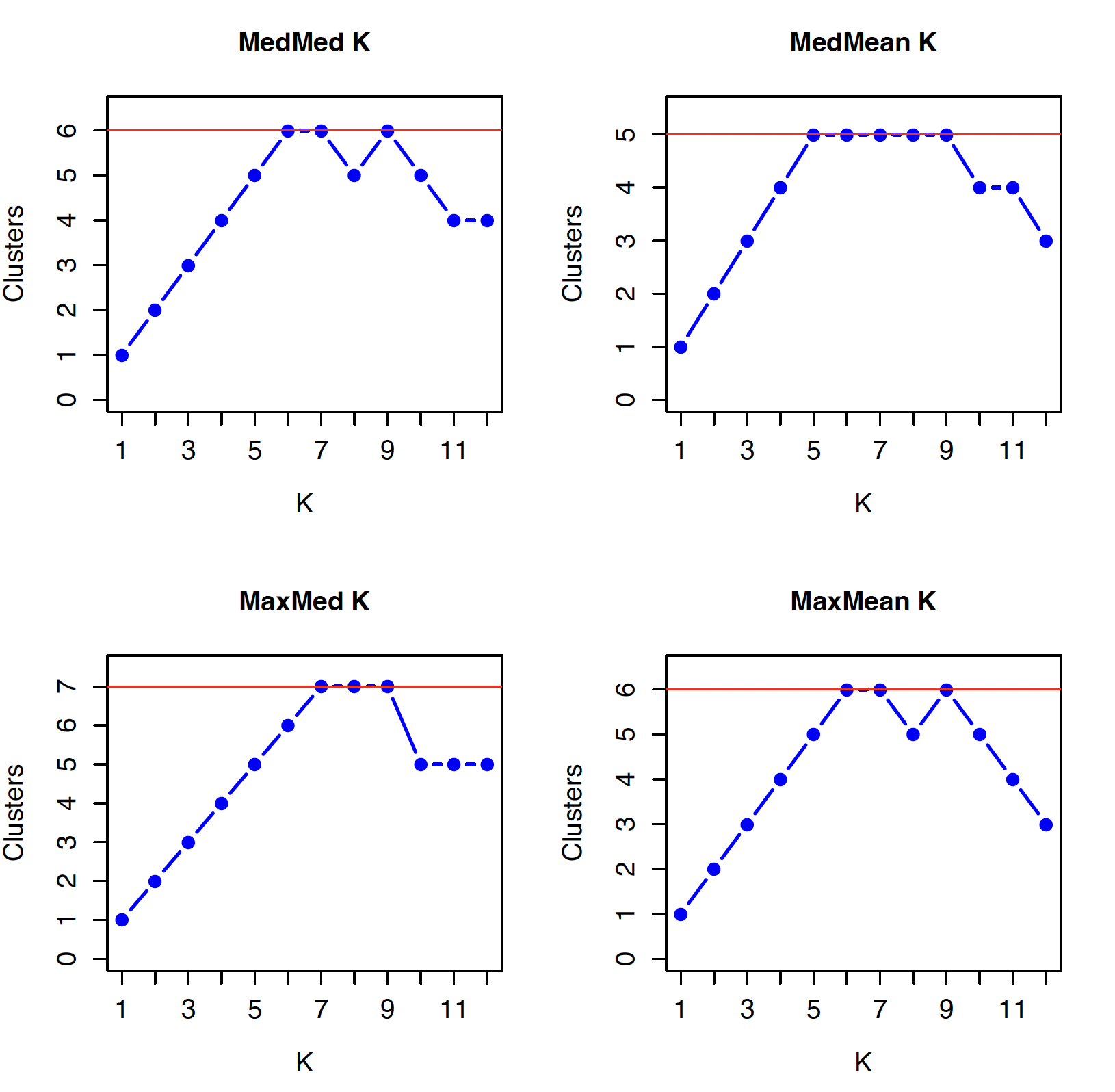

Supplement: Supplementary file 1 [file animals-13-02139-s001.zip › Figure S1.jpeg]

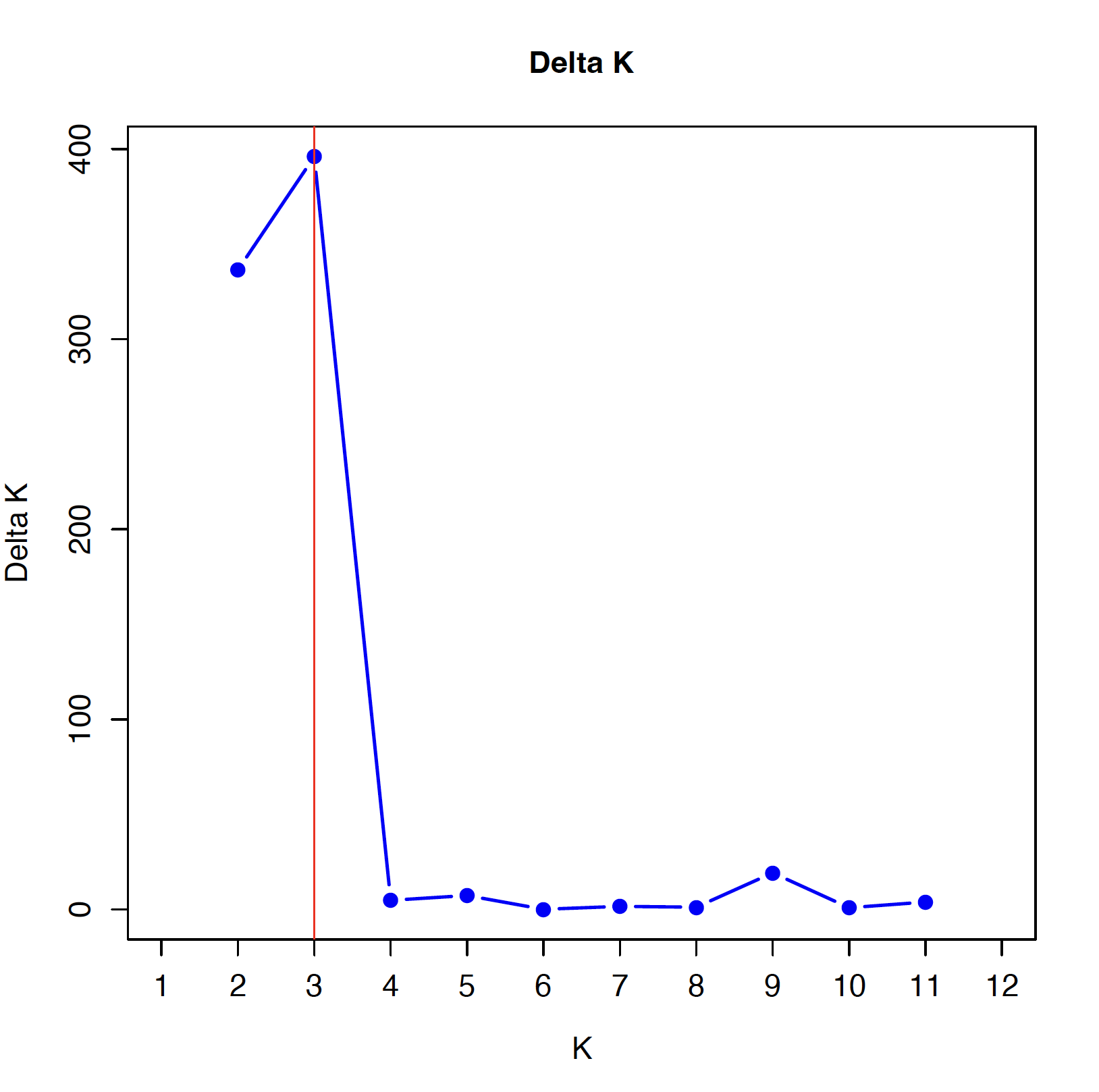

Supplement: Supplementary file 1 [file animals-13-02139-s001.zip › Figure S2.jpeg]

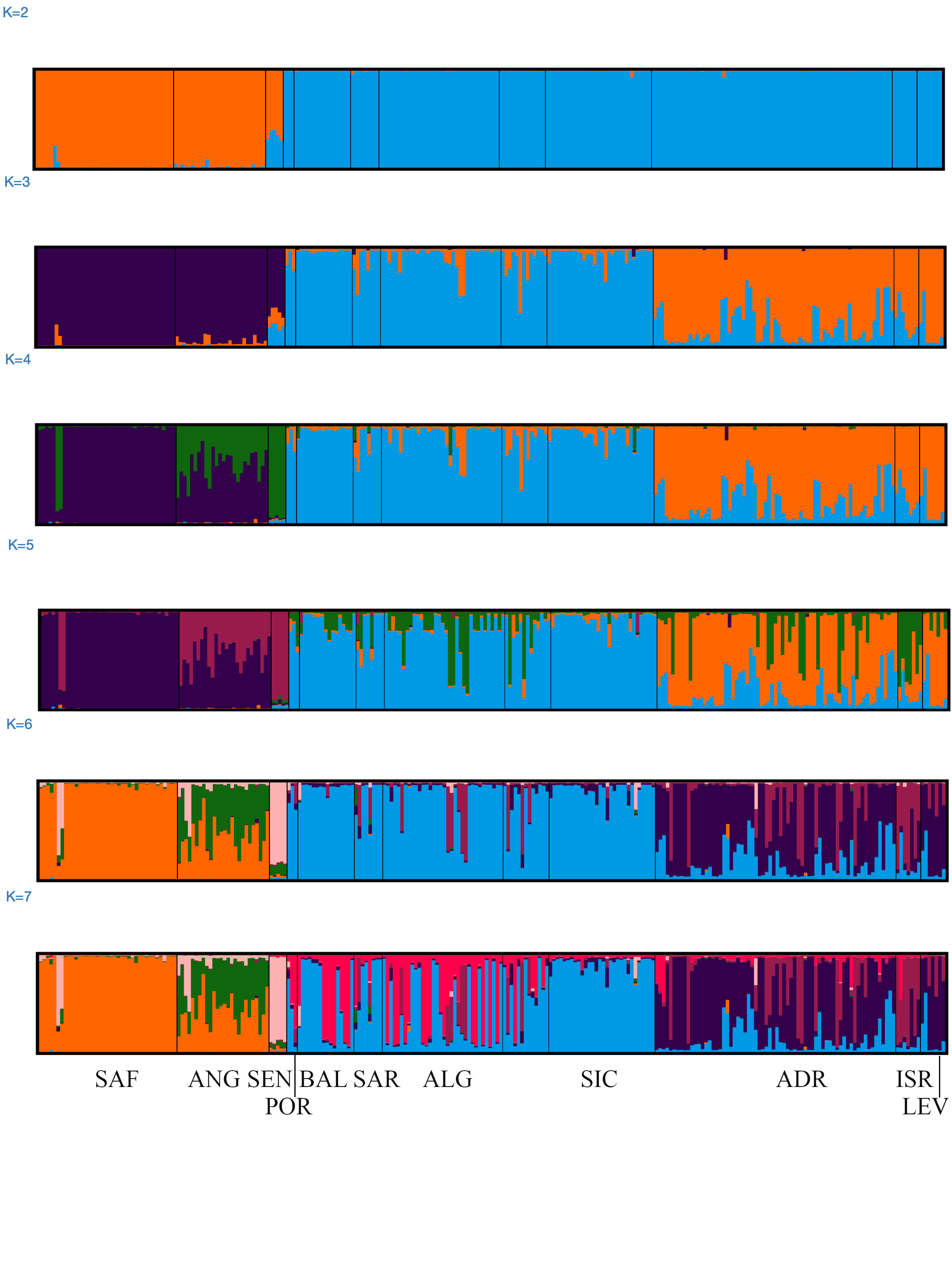

Supplement: Supplementary file 1 [file animals-13-02139-s001.zip › Figure S3.jpeg]

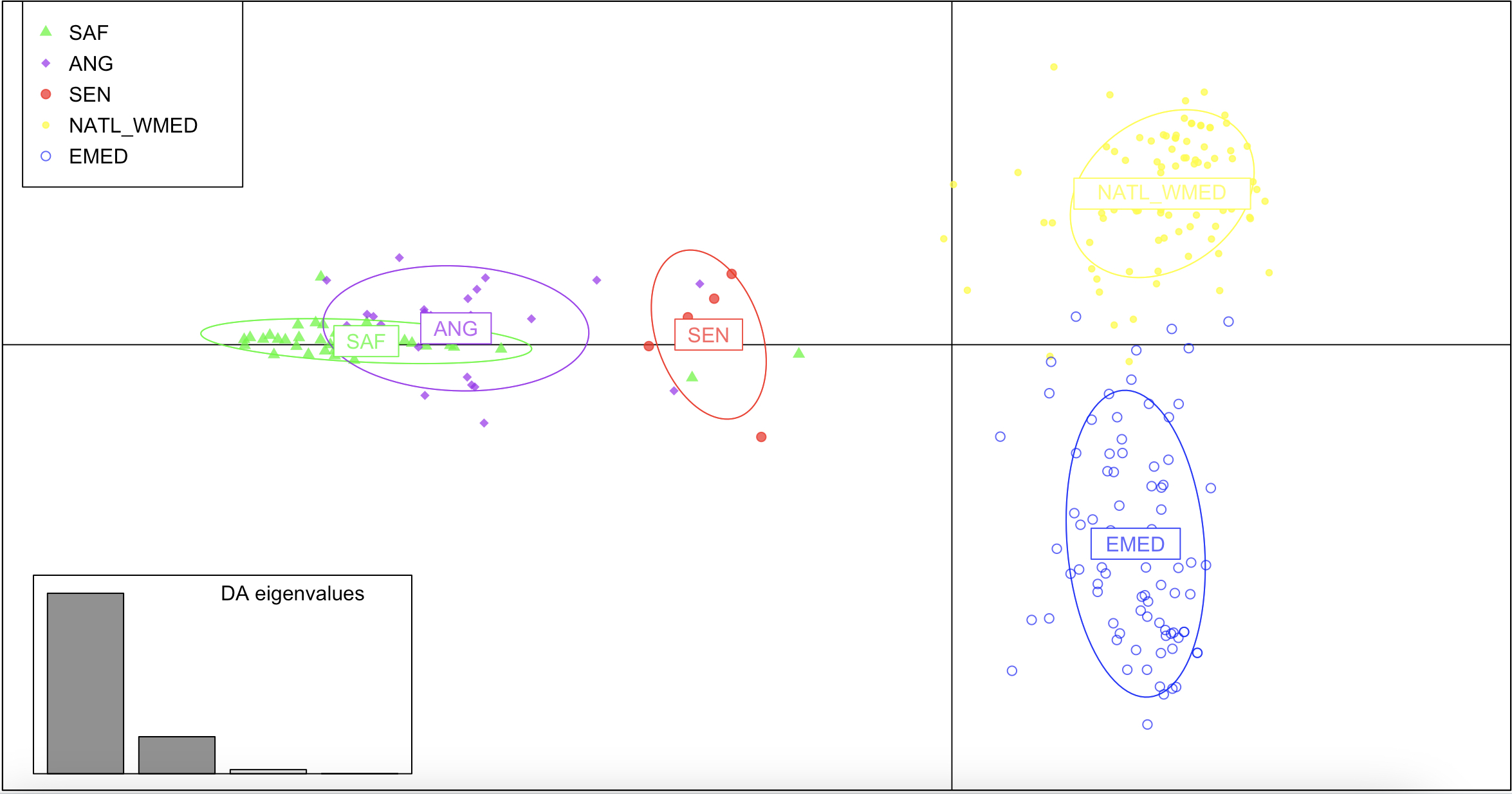

Supplement: Supplementary file 1 [file animals-13-02139-s001.zip › Figure S4.JPEG]
